# Trust in Institutions and the COVID-19 Threat: A Cross-Sectional Study on the Public Perception of Official Recommendations and of Othering in Switzerland

**DOI:** 10.3389/ijph.2021.1604223

**Published:** 2022-01-10

**Authors:** Ingrid Gilles, Marie-Annick Le Pogam, Margaux Perriraz, Adrian Bangerter, Eva G. T. Green, Christian Staerklé, Franciska Krings, Pascal Wagner-Egger, Isabelle Peytremann-Bridevaux

**Affiliations:** ^1^ Center for Primary Care and Public Health, University of Lausanne, Lausanne, Switzerland; ^2^ Institute of Work and Organizational Psychology, University of Neuchâtel, Neuchâtel, Switzerland; ^3^ Institute of Psychology, University of Lausanne, Lausanne, Switzerland; ^4^ Faculty of Business and Economics, University of Lausanne, Lausanne, Switzerland; ^5^ Department of Psychology, University of Fribourg, Fribourg, Switzerland

**Keywords:** COVID-19, vaccination intent, official recommendations, trust in institutions, disease threat, othering

## Abstract

**Objectives:** To explore how perceived disease threat and trust in institutions relate to vaccination intent, perceived effectiveness of official recommendations, and to othering strategies.

**Methods:** We conducted a cross-sectional survey of Swiss adults in July 2020. Outcome variables were vaccination intent, perceived effectiveness of official recommendations and othering strategies (labelling a given social group as responsible for the disease and distancing from it). Independent variables were perceived disease threat, trust in various institutions, perceived health-related measures, and sociodemographic variables. Linear and logistic regressions were performed.

**Results:** The response rate was 20.2% (1518/7500). Perceived disease threat and trust in medical/scientific institutions were positively associated with vaccination intent and perceived effectiveness of official recommendations for coronavirus mitigation measures. Only disease threat was associated with a perception of effectiveness among othering strategies. Age and education levels were associated with vaccination intent.

**Conclusion:** Reinforcing trust in medical/scientific institutions can help strengthen the perceived effectiveness of official recommendations and vaccination. It however does not prevent adherence to ineffective protecting measures such as othering strategies, where decreasing perceptions of epidemic threat appears to be more efficient.

## Introduction

Since the emergence of the first cases of COVID-19 in December 2019, an unprecedented global health crisis has rapidly evolved, with more than 239 million people infected and almost 4.9 million deaths worldwide reported in fall 2021 [1]. In the absence of an available treatment and until a vaccine was developed, most countries adopted population-oriented preventive strategies to contain corona-virus spread and avoid overwhelming their health services. These strategies, resulting in official recommendations or regulations, ranged from basic hygiene measures (e.g., regular hand washing, use of hand sanitizer when hand washing is not possible, sneezing/coughing into a paper tissue or the elbow) and the adoption of new practices (i.e., wearing a facemask) to more stringent measures, such as quarantine, confinement, and limitation of social interactions. However, the success of these strategies depends on a critical factor—public support and compliance with official recommendations.

Public compliance with official recommendations depends on individual, social and systemic factors. For example, results of studies conducted in the COVID-19 context showed that women and individuals with a higher level of education were more compliant with recommendations [2]. At the system level, official communications that used moral advice were poorly effective [3], whereas transparent communication induced more support from the public [4]. Trust in institutions and perceptions of virus threat emerged as important determinants of compliance [5–7]. Individuals who perceived the virus as a serious threat tended to comply with official measures [5], whereas those who considered that the disease was not dangerous tended to follow recommendations less strictly [8]. Similarly, people who trusted medical and scientific institutions tended to adhere more strongly to official recommendations, whereas weak trust was associate with potential non-compliance [7, 9].

Attention has also been paid to public compliance with vaccination [10], which is proposed as the most effective way to contain COVID-19 [11]. In Switzerland, vaccination outside epidemic periods is relatively well accepted, but the coverage depends on the disease (e.g., 96% of 16-year-old adolescents are completely vaccinated against measles, but only 59% of 16-year-old girls with two doses of human papilloma virus vaccine [12]) and on other factors such as political or community opinions [13]. Acceptability of vaccination is thus crucial and several studies have highlighted scepticism among the population towards the COVID-19 vaccine, mainly for safety reasons [14]. Once again, perceived threat and trust in institutions have been shown to favour vaccine acceptance [15, 16].

To fully understand the public response to official recommendations or vaccination, it is necessary to identify not only the factors that lead to this response, but also the underlying mechanisms by which individuals perceive and understand the disease. For example, Wong and Jensen [17] found that high levels of trust in the government could paradoxically lead to less compliance if the public perceived the pandemic situation as low risk and under government control. The literature on public understanding of emerging infectious disease has shown that in the absence of pre-existing knowledge, people try to make sense of the new disease by constructing common-sense explanations that may not be rational or correct from a public health perspective [18]. These explanations, collectively constructed through interpersonal, internet and media communication, help individuals to cope with the uncertainty of the new situation and guide their actions [19]. In addition, they may lead people to adopt protective strategies that are not objectively effective. One of these strategies, referred to as “othering” [20], consists of distancing oneself from the disease by associating it to a specific group perceived to be the source of the disease or responsible for its spread [21]. This process consists of marginalization and social exclusion of the “othered” groups [22] notably, by the belief that avoiding the specific group could prevent them from getting ill and thus act as a protective strategy against the disease [23]. This phenomenon has been observed in the context of health care [24, 25] and is also relevant in epidemic situations [26]. As an example, recent research in the current COVID-19 pandemic has revealed the identification of people of Asian descent [27] or healthcare professionals [28] as disease vectors. Attributing effectiveness to such strategies of distancing could lead people to comply less with official recommendations, including vaccination [29]. It is therefore critical to understand how factors associated with compliance (i.e., perception of disease threat and trust in institutions) associate with the perceived effectiveness of official recommendations, including vaccination intent, and othering strategies.

The aim of our study was to explore how the perceived threat of the virus and trust in different institutions relate to vaccination intent, to perceived effectiveness of official recommendations and of ineffective protective measures such as othering strategies.

## Methods

### Study Design and Participants

We conducted a cross-sectional study among 7,500 Swiss adults residing in three cantons of French-speaking Switzerland in July 2020 after the first wave of COVID-19 using a self-report questionnaire. Contacted persons were randomly selected from a database of 517,000 addresses according to gender (50% women), canton of residence (one-third of the sample per canton), and age (50% < 65 years). To increase the response rate, we opted for a dual strategy: paper questionnaires were sent to individuals aged 65 years and older and online questionnaires were sent to those <65 years. Online questionnaires have several advantages, such as convenience for respondents, flexibility in designing the questionnaire and timeliness of responses [30], but they also include limitations. The most problematic consists of a lower access to numeric tools by some groups of respondents, such as the elderly [30, 31]. No reminders were sent.

### Measures

The items were drawn from a questionnaire used in the context of a study on public understanding of the H1N1 outbreak and reported in several publications [15, 26, 32, 33]. In the present study, questions were slightly adapted to match with the COVID-19 pandemic.

#### Outcome Variables

The two outcomes were vaccination intent and perceived effectiveness of protective measures. Vaccination intent was assessed with the following single item: “If an effective vaccine against the coronavirus was made available to the population in the future, would you get vaccinated?” The response format was dichotomous (0 = no; 1 = yes). The perceived effectiveness of protective measures consisted of two dimensions. First, we asked respondents to assess, the effectiveness of five official protective measures recommended by authorities (2-meter distance, wearing a facemask, washing hands regularly, sneezing into the elbow, avoiding kisses and handshakes; Cronbach’s alpha = 0.71) on a 5-point Likert scale (1 = not effective at all to 5 = totally effective). We then asked respondents to assess the effectiveness of two othering strategies using the same 5-point Likert scale, limiting personal contacts with foreigners and with people not paying attention to their hygiene; (Pearson’s *r* = 0.32, *p* = 0.001). Mean scores for effectiveness were computed for both dimensions (official protective measures and othering strategies).

#### Main Groups of Independent Variables

Trust in institutions and perceptions about the disease represent the two main groups of independent variables.

Trust in three categories of institutions was assessed using a 5-point Likert scale. 1) medical/scientific institutions (medicine, research, hospitals and pharmacies; Cronbach’s alpha = 0.79); 2) governmental (federal and cantonal governments; Pearson’s *r* = 0.71); 3) non-Swiss institutions [European Union (EU), foreign governments, World Health Organization (WHO); Cronbach’s alpha = 0.77]. We computed a mean trust score for each institution category.

Perceptions about the disease included the following variables: disease threat concerns about social and economic consequences; “perceived infectability” [34]; and the belief that life will return to normal after the pandemic.

Disease threat was assessed with four items that asked participants to rate if the coronavirus represented a threat to them personally, their relatives, the country, and humanity on a 5-point Likert scale (1 = not at all to 5 = yes, totally). We computed a global perceived mean threat score from these four items (Cronbach’s alpha = 0.86).

Concerns about the social and economic consequences of the disease were assessed with four items. We asked participants to state, on a 5-point Likert scale, whether they felt concerned about the consequences of the pandemic for the Swiss economy, their own economic situation, society in the coming years, and their own future. A global concern mean score was computed from these four items (Cronbach’s alpha = 0.74).

“perceived infectability” was measured by using four items from the Perceived Vulnerability to Disease Scale [34]. This subscale assesses individuals’ general beliefs regarding their susceptibility to being infected by viruses or contracting an infectious disease (5-point Likert scale; 1 = not at all susceptible to 5 = very susceptible). A “perceived infectability” mean score was computed from the four items (Cronbach’s alpha = 0.71).

We also measured the belief that life will return to normal after the pandemic with a single item specifically constructed for this study. Participants had to rate their agreement with the following statement on a 5-point Likert scale (1 = totally disagree to 5 = totally agree): “After the pandemic, my life will quickly return to the way it was before.”

#### Confounding and Sociodemographic Variables

We considered several variables related to the respondents’ health status: life satisfaction (single item on a 5-point Likert scale) [35]; perceived health (single item on a 5-point Likert scale) [36]; being affected by one or more chronic diseases (single item, yes vs. no); having been in contact with a person affected by COVID-19 (single item, yes vs. no); and having had symptoms consistent with COVID-19 (single item; yes confirmed by a positive test, yes but with a negative test, yes but no test done, no symptoms).

The following sociodemographic variables were included at the end of the questionnaire: gender (men/women), age (continuous), household size (number), education level (secondary, high school, college or university), standard of living (Likert scale from 1 = very poor to 6 = very high), political orientation (11-point scale from 0 = left to 10 = right).

### Data Analysis

Apart from for vaccination intent (dichotomous variable) and where specified, we treated all variables as continuous after checking for distribution linearity and normality.

We first conducted descriptive analyses to characterize respondents. We then performed multiple logistic regressions to explain vaccination intent and multiple linear regressions to explore the perceived effectiveness of protective measures (official protective measures and othering strategies). Independent variables were entered by block. Block 1: demographic variables; block 2: health-related variables; block 3: beliefs about health issues and the coronavirus (disease threat, concerns about social and economic consequences of the disease, “perceived infectability,” and return to a normal life after the pandemic); block 4: variables measuring trust in institutions (medical/scientific institutions, the Swiss government and non-Swiss institutions). Results presented below concern only block 4 (see Supplementary Material for detailed results). We checked multicollinearity between predictive variables by using the variance inflation factor and tolerance indicators. As the missing value rate was low (<1.0% for the outcomes and main independent variables), we did not impute missing values and opted for a listwise deletion procedure. Analyses were conducted using IBM SPSS Version 26.0 (SPSS 2020).

## Results

### Descriptive Analyses

Of 7,500 persons invited to participate, 1,518 completed the questionnaire (20.2% response rate). Respondent characteristics are shown in Table 1 (women, 50%; <65 years, 50%). Approximately one-half of respondents had a university degree and almost 40% had terminated their studies after secondary school level. The standard of living was relatively high and most respondents reported that they were satisfied with their lives. More than 90% of respondents reported good-to-excellent health status, with approximately one-third having a chronic condition. One percent of respondents reported a confirmed case of COVID-19 and 8% reported symptoms compatible with COVID-19, but without having had a test. Although the mean age was higher in our study compared to the Swiss adult population [mean age, 42.5 years; 2019 Federal Office of Statistics (FOS) data], our sample was similar in terms of the number of people living in a household (2.2; 2019 FOS data) and life satisfaction (approximately 7.5 on a scale from 0 to 10 according to 2017 Organisation for Economic Co-operation and Development data). Our sample also included more people with low levels of education compared to the Swiss population [≈11.0%; 2019 Federal Office of Statistics (FOS) data].

**TABLE 1:** Characteristics of respondents (Trust, disease threat and protective measures, Switzerland, 2020).

	All (*n* = 1518)
Sociodemographic and context variable	
Age, mean (SD)	61.7 (14.9)
Sex, female	50.3%
Number of persons in the household, mean (SD)	2.4 (1.4)
Education level	
Secondary	39.8%
High school	13.8%
College/university degree	46.4%
Standard of living, mean (SD)	4.0 (0.7)
Self-reported health, good-to-excellent	93.1%
Satisfaction with life, mean (SD)	4.2 (0.7)
Chronic diseases, yes	31.5%
Contact with a COVID-19 patient, yes	15.8%
COVID-19 symptoms	
Yes, confirmed by a test	1.1%
Yes, but with a negative test	3.6%
Yes, but no test done	8.0%
No symptoms	87.4%
Main group of independent variables	
Disease threat, mean (SD)	4.0 (0.9)
Concern for the future, mean (SD)	3.4 (0.8)
“perceived infectability”, mean (SD)	2.4 (0.8)
Return to a normal life after the pandemic, mean (SD)	3.1 (1.1)
Trust in medical/scientific institutions, mean (SD)	3.9 (0.7)
Trust in the Swiss government, mean (SD)	4.1 (0.7)
Trust in non-Swiss institutions, mean (SD)	3.2 (0.8)
Outcome variables	
Perceived effectiveness of official measures, mean (SD)	4.3 (0.5)
Perceived effectiveness othering, mean (SD)	3.3 (0.9)
Vaccination intent, yes	76.0%

Approximately one-sixth of respondents declared that they were in contact with a patient with COVID-19 at the time of the survey. About two thirds felt “somewhat seriously” to “seriously” threatened by COVID-19, and one-half reported a fair-to-strong level of trust in medical/scientific institutions. Concerning vaccination intent (Table 1), results showed that three-quarters of respondents were willing to accept vaccination after the first wave of COVID-19.

**FIGURE 1:**
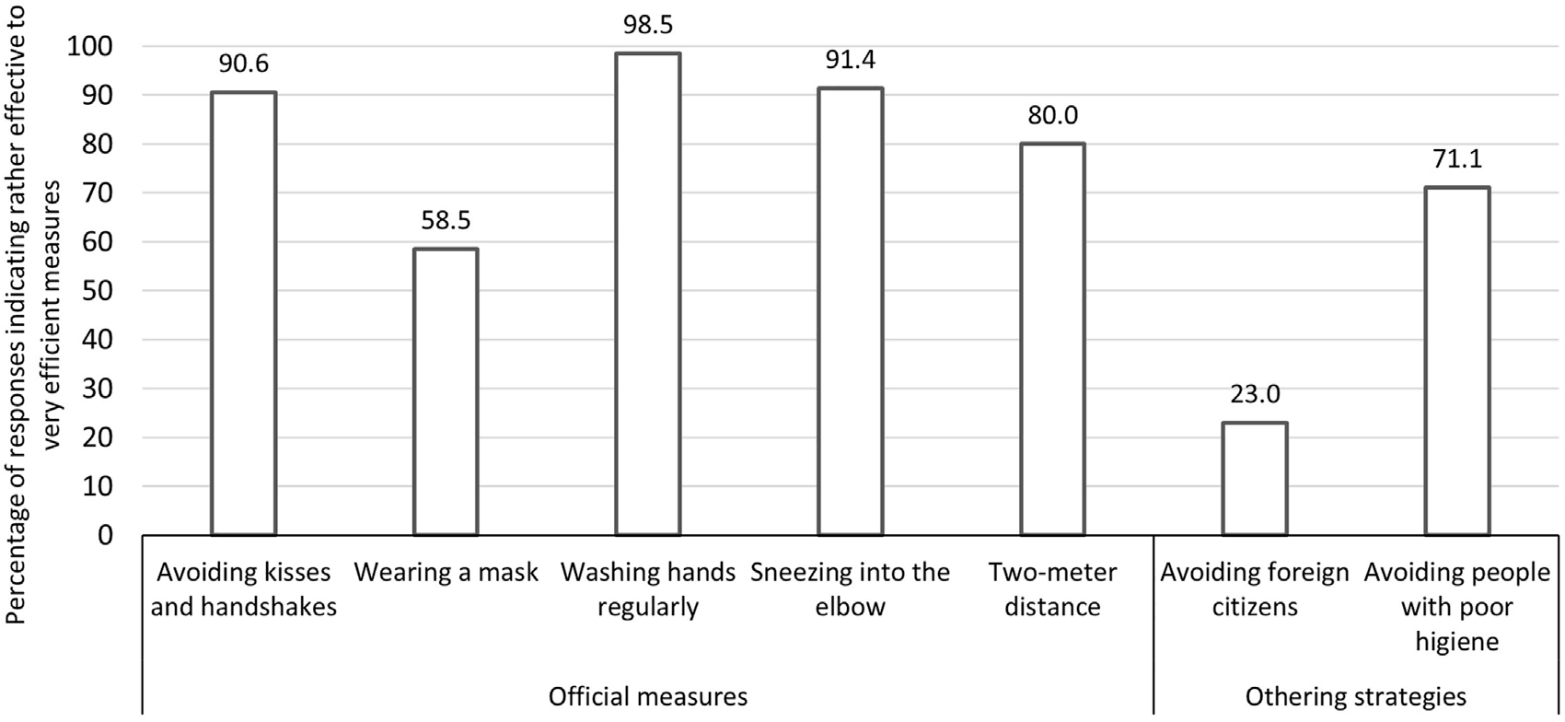
Percentage of respondents estimating that the different official protective measures and othering strategies were rather effective or very effective (Trust, disease threat and protective measures, Switzerland, 2020).

Concerning official protective measures, 80.0% of respondents perceived them as “rather effective” to “very effective.” The most effective measures were considered to be regular hand washing (98.5% considered it “rather effective” to “very effective”) and sneezing into the elbow (91.4% considered it “rather effective” to “very effective”). Wearing a facemask was considered as the least effective measure, with only 57.9% of respondents attributing effectiveness to this measure (see Figure 1).

Regarding othering strategies, 31.7% of respondents perceived them to be “rather effective” to “very effective.” More specifically, respondents judged avoiding people with poor hygiene to be more effective (71.1% considered it to be “rather effective” to “very effective”) than avoiding personal contacts with foreign citizens (23.0% considered it “rather effective” to “very effective”) (see Figure 1).

### Regression Analyses Results

#### Vaccination Intent

Logistic regression analysis (Table 2) showed that age, level of education, disease threat, “perceived infectability,” and trust in medical/scientific institutions were the variables most associated with vaccination intent. Respondents with a higher education were almost two times more likely to accept vaccination. For respondents reporting a high level of threat and of “perceived infectability,” these odds ratios were 1.6 and 1.4, respectively. Trust in medical/scientific institutions was most associated with vaccination intent. Respondents who reported higher trust were 2.5 times more likely to accept vaccination than those who reported a weaker trust.

**TABLE 2:** Multiple linear and logistic regression analyses with perceived effectiveness of official protective measures, vaccination intent and othering strategies as outcomes (Trust, disease threat and protective measures, Switzerland, 2020).

Variable	Vaccination intent		Effectiveness of official protective measures		Effectiveness of othering strategies	
	OR	95% CI	B	95% CI	B	95% CI
Age	1.02**	1.01 to 1.03	0.00	−0.00 to 0.01	0.01**	0.00 to 0.01
Gender, women	0.67	0.47 to 0.95	0.13***	0.07 to 0.20	0.05	−0.06 to 0.16
Number of persons in the household	1.08	0.93 to 1.25	0.00	−0.02 to 0.03	0.00	−0.04 to 0.04
Education level, higher education	1.74**	1.21 to 2.50	0.02	−0.04 to 0.08	−0.18**	−0.29 to −0.07
Standard of living	1.25	0.95 to 1.65	0.03	−0.01 to 0.08	−0.12**	−0.21 to −0.04
Political orientation	1.01	0.94 to 1.10	0.01	−0.01 to 0.02	0.11***	0.09 to 0.13
Satisfaction with life	1.11	0.84 to 1.46	0.05	−0.00 to 0.10	−0.06	−0.14 to 0.03
Self-reported health	0.95	0.71 to 1.27	0.07**	0.02 to 0.12	0.08	−0.01 to 0.17
Chronic conditions, yes	0.89	0.57 to 1.37	0.06	−0.02 to 0.13	0.07	−0.06 to 0.20
Contact with a COVID-19 patient, yes	0.87	0.57 to 1.34	0.01	−0.07 to 0.09	−0.04	−0.18 to 0.10
Disease threat	1.63***	1.34 to 2.00	0.13***	0.10 to 0.17	0.25***	0.18 to 0.31
Concern for the future	1.25	0.99 to 1.56	0.02	−0.02 to 0.06	0.02	−0.06 to 0.09
“Perceived infectability”	1.44**	1.12 to 1.84	0.05	0.01 to 0.09	0.09	0.02 to 0.16
Return to a normal life after the pandemic	1.09	0.92 to 1.28	−0.04	−0.06 to −0.01	−0.05	−0.10 to 0.01
Trust in medical/scientific institutions	2.46***	1.75 to 3.46	0.14***	0.08 to 0.20	−0.00	−0.10 to 0.10
Trust in the Swiss government	0.89	0.66 to 1.21	0.02	−0.04 to 0.07	−0.05	−0.15 to 0.05
Trust in non-Swiss institutions	1.10	0.85 to 1.43	0.01	−0.04 to 0.05	−0.04	−0.11 to 0,04
*R* ^2^	0.25		0.17		0.23	

**p < 0.01; ***p < 0.001.

OR, odds ratio; CI, confidence interval.

#### Official Protective Measures

Linear regression analyses (Table 2) showed that women and respondents reporting a good health status perceived official protective measures as more effective than respectively men and respondents with a poorer health status. Disease threat and trust in medical/scientific institutions were also significantly associated with perceived effectiveness of official protective measures: the more respondents felt threatened by the virus and the more they trusted medical/scientific institutions, the more they perceived official protective measures as effective.

#### Othering Strategies

Linear regression analysis on the effectiveness of othering strategies indicated that older respondents, those less educated with a lower standard of living or adhering to right-wing political ideologies attributed more effectiveness to othering strategies than other respondents. Moreover, disease threat was strongly associated with othering strategies: the more respondents felt threatened by the virus, the more they attributed effectiveness strategies consisting of avoiding some specific social groups.

Table 2 presents the results of the last block of regression models; details of the four consecutive blocks are shown in Supplementary Table S1.

## Discussion

In this study, we aimed to investigate the association between threat and trust in different institutions with vaccination intent, the perceived effectiveness of official recommendations, and also with non-effective measures such as othering strategies. Our results confirm the central role of threat perception and institutional trust in the perception of official protective measures and vaccination intent. They also reveal that in July 2020 (i.e., after the first wave of COVID-19), almost one-third of respondents perceived othering strategies “rather efficient” to “very efficient,” and disease threat was strongly associated with othering strategies whereas trust was not.

Concerning othering strategies, we found that avoiding people with a poor hygiene was perceived as effective by almost three-quarters of respondents in summer 2020. Historically, othering has been used as a protective strategy against infectious diseases [37]. Blaming or locating the spread of disease in a group that is not one’s own is a mean to symbolically remove the threat of the disease [38]. It also allows people to believe that they are less likely to contract the disease if they interact only with people in their close circle despite the fact that main clusters have been identified in families [39]. Othering strategies are closely related to stigmatization processes: by assimilating a given group with the disease and blaming it for its spread, people identify the “othered groups” as the deviant group, which is thus stereotyped and discredited [40]. Such stigmatization processes have been observed in the COVID-19 pandemic context [27, 28, 41]. For example, in a United States national survey conducted during the first year of the pandemic, 40% of respondents expressed their willingness to engage in discriminatory behavior towards Asian people [41]. In another study focusing on avoidance of healthcare professionals, 25% of US and Canadian respondents agreed with the statement that “for the safety of the community, healthcare workers should not go out in public” [28].

In our study, avoiding people with poor hygiene was perceived more effective than facemasks and a little less effective than keeping a two-meter distance between people. Our findings concerning the facemask might be due to the fact that in July 2020 in Switzerland, its use was not generalized and restricted to stores and public transport. However, this was not the case for the two-meter distance measure, which had been recommended since the beginning of the pandemic. As the perceived effectiveness of protective behavior is strongly related to the choice to adopt a protective behavior or not [41], our results suggest that it is important for authorities to ensure not only that the public perceives official measures (including vaccination) as effective, but also that it perceives other protective strategies, such as othering, as ineffective [41]. Therefore, communicating on both the effectiveness of protective measures and the ineffectiveness of othering strategies seems essential. For example, public health authorities could reinforce the message that reducing private gatherings or gatherings in the public space is a more effective protection strategy than avoiding some specific groups of people symbolically associated with the disease (e.g., foreigners, homeless people).

The main factor associated with othering strategies was disease threat, which is coherent with past research [26, 37, 42, 43]. The status of this variable was interesting. Indeed, it was associated with both more perceived effectiveness of official protective measures and greater vaccination intent as observed in other outbreaks [15], but also with more perceived effectiveness of othering strategies. In other words, feeling threatened by the virus can lead people to get vaccinated and conform to official recommendations, but it can also lead people to adopt objectively inefficient strategies. The latter result is important in terms of communication, as raising fear is known to be useful to lead people to adopt healthy behaviors [44]. Thus, according to our findings, activating the disease threat among people may lead them to adopt recommended measures. However, this can also lead to counterproductive reactions such as denial, anxiety, increased risk behavior or, as observed in our study, adherence to false beliefs, such as group avoidance strategies [45, 46] which disrupt social cohesion [47]. A meta-analysis conducted on the use of fear appeals in health campaigns found that this counterproductive effect can be reduced by giving people the confidence that they are able to perform the recommended behavior [48]. For example, the authors suggest that this could be achieved by targeting concrete barriers people encounter to practice the recommended behavior in communication messages and addressing cues to overcome them [48]. This strategy could also be very relevant in the context of COVID-19 [49].

Concerning trust in medical/scientific institutions, our results confirm the positive association between trust and compliance with official protective measures and vaccination [15, 50]. Nevertheless, they also suggest that increasing trust in an institution globally is not a sufficient strategy to prevent people from perceiving objectively ineffective protective strategies as effective. This result differs from Dhanani and Franz who found that trust in science was associated with less stigmatization during the COVID-19 pandemic [41]. However, in the latter report, the mean age was quite low compared to our study and more than two-thirds of respondents had a college or a university degree (only 46.4% in our study), two factors we found attenuating the perceived effectiveness of othering strategies. Thus, the association between trust in science and othering strategies may be moderated by such factors. Future studies should examine whether strengthening trust in medical/scientific institutions encourages people to respect public health recommendations, undergo vaccination, and reduce discriminatory behavior [41], but only in specific populations, such as the younger or higher educated.

This latter point also concerns the final important result of our study regarding the role of sociodemographic variables to understand compliance with vaccination and the othering phenomenon, particularly education or standard of living. Indeed, the extant literature suggests that adhering to official recommendations is easier for people living in safe social conditions [51, 52]. The fact that most constraints imposed by official recommendations (quarantine, remote working, and reduction of professional activities) affected people’s work could explain why these socioeconomic factors appear to be significant. To encourage compliance with official recommendations, including vaccination, support from authorities or communities should be implemented and/or enhanced and sustained by a long-term compensation system (e.g., in the case of lost wages) [53].

### Strengths and Limitations

Our study has several strengths, such as the large sample size and the fact that we explored both compliance with official recommendations and symbolic strategies, such as othering*.* However, several limitations need to be considered when interpreting the results. First, our sample is not representative of the Swiss population in terms of age: 50% of respondents consisted of people 65 years and over, although they represent approximately only one-fifth of the Swiss population. Apart from age, the characteristics of our sample were similar to those of the Swiss population (gender, household size, and satisfaction with life according to 2017 and 2019 data from the FOS and the Organisation for Economic Co-operation and Development). Nevertheless, the difference in age structure may limit the generalizability of our results. In addition, generalization to other countries may also be limited as health systems and overall contexts vary, similar to COVID-19 containment strategies across regions. However, our findings on disease threat and trust are coherent with those published in similar studies strengthening our confidence in our results. . Second, the response rate may be considered to be low. This rate is probably underestimated for the electronic administration mode of the survey as not all email addresses were valid. This is a recognized problem in electronic surveys targeting the general population [30]. It is therefore possible that a substantial number of people we contacted did not receive the invitation to participate. That said, our response rate is similar to international population-based studies, such as the International Health Policy Survey of the Commonwealth Fund, which reported an overall response rate of 22% [54]. Third, the cross-sectional nature of our study prevents firm conclusions in terms of causality. However, our results are consistent with those reported in a longitudinal study during the H1N1 outbreak [15] or more recently during the COVID-19 pandemic in cross-sections studies [55].

### Conclusion

As large-scale vaccination campaigns may have difficulties to spread in Switzerland, it is crucial to consider trust in medical/scientific institutions and perceived disease threat to improve public compliance with vaccination and official recommendations. Clear and transparent communication, including timely explanations about official strategies, is key to avoid incoherencies or uncertainties. It is also important for authorities to better understand the psychological processes that may lead people to adopt non-evidence-based strategies, such as othering strategies, and to consider these processes when planning vaccination strategies.

## Data Availability

Data supporting the findings of this study are available from the corresponding author, IG, upon reasonable request.
