# Supplementary material for: Trust in Institutions and the COVID-19 Threat: A Cross-Sectional Study on the Public Perception of Official Recommendations and of Othering in Switzerland
Source: Int J Public Health. 2022 Jan 10;66:1604223. doi: 10.3389/ijph.2021.1604223 (PMC8790817; doi:10.3389/ijph.2021.1604223)
Supplement: Supplementary file 1 [file DataSheet1.doc]

Detailed results by blocks for linear and logistic regressions

S1. Detailed multiple linear regression analysis with vaccination intent overall (Trust, disease threat and protective measures, Switzerland, 2020).	
Model			     95% C.I.	
	B	Std. Error	OR	Lower	Upper	
1							
	Age	0.027	0.007	1.028	1.015	1.041	
	Gender. women	-0.403	0.168	0.668	0.481	.928	
	Number of persons in the household	0.037	0.068	1.038	0.909	1.185	
	Education level. higher education	0.384	0.168	1.469	1.057	2.040	
	Standard of living	0.223	0.123	1.250	0.983	1.589	
	Political position	0.021	0.035	1.021	0.952	1.094	
2							
	Age	0.022	0.007	1.022	1.008	1.035	
	Gender. women	-0.393	0.169	0.675	0.484	.941	
	Number of persons in the household	0.032	0.068	1.033	0.904	1.179	
	Education level. higher education	0.440	0.170	1.553	1.112	2.169	
	Standard of living	0.243	0.131	1.275	0.987	1.648	
	Political position	0.027	0.036	1.028	0.958	1.102	
	Satisfaction with life	0.179	0.130	1.197	0.928	1.542	
	Self-reported health	-0.308	0.125	0.735	0.575	.940	
	Chronic conditions. yes	0.056	0.204	1.058	0.709	1.579	
	Contact with a COVID-19 patient. yes	-0.306	0.200	0.737	0.498	1.090	
3							
	Age	0.018	0.007	1.018	1.004	1.032	
	Gender. women	-0.400	0.177	0.670	0.474	.948	
	Number of persons in the household	0.042	0.071	1.043	0.908	1.198	
	Education level. higher education	0.599	0.181	1.820	1.276	2.597	
	Standard of living	0.297	0.137	1.346	1.030	1.760	
	Political position	0.014	0.038	1.014	0.941	1.092	
	Satisfaction with life	0.208	0.138	1.231	0.940	1.613	
	Self-reported health	-0.013	0.143	0.987	0.746	1.307	
	Chronic conditions. yes	-0.066	0.213	0.936	0.616	1.423	
	Contact with a COVID-19 patient. yes	-0.109	0.212	0.896	0.591	1.359	
	Disease threat 	0.635	0.098	1.888	1.557	2.288	
	Concern for the future 	0.193	0.116	1.213	0.965	1.523	
	Perceived infectability 	0.335	0.122	1.398	1.102	1.775	
	Return to a normal life after the pandemic	0.105	0.081	1.111	0.947	1.303	
4							
	Age	0.019	0.007	1.019	1.005	1.034	
	Gender. women	-0.406	0.182	0.667	0.467	.952	
	Number of persons in the household	0.075	0.074	1.078	0.933	1.246	
	Education level. higher education	0.552	0.186	1.736	1.206	2.500	
	Standard of living	0.225	0.140	1.253	0.951	1.650	
	Political position	0.013	0.040	1.013	0.938	1.095	
	Satisfaction with life	0.100	0.142	1.105	0.835	1.461	
	Self-reported health	-0.050	0.147	0.951	0.713	1.269	
	Chronic conditions. yes	-0.122	0.221	0.885	0.574	1.366	
	Contact with a COVID-19 patient. yes	-0.139	0.218	0.870	0.567	1.335	
	Disease threat 	0.491	0.103	1.634	1.336	1.999	
	Concern for the future 	0.225	0.120	1.252	0.989	1.585	
	Perceived infectability 	0.363	0.127	1.438	1.122	1.842	
	Return to a normal life after the pandemic	0.082	0.085	1.085	0.920	1.281	
	Trust in medical and scientific institutions 	0.900	0.174	2.458	1.749	3.456	
	Trust in the Swiss government 	-0.117	0.156	0.890	0.656	1.207	
	Trust in non-Swiss institutions 	0.097	0.131	1.101	0.852	1.425	


S2. Detailed multiple linear regression analysis with effectiveness of official protective measures overall (Trust, disease threat and protective measures, Switzerland, 2020).	
Model	Unstandardized Coefficients	Standardized Coefficients	t	P Value	
	B	Std. Error	Beta			
1							
	Age	0.004	0.001	0.125	3.342	0.001	
	Gender, women	0.136	0.033	0.135	4.075	0.000	
	Number of persons in the household	-0.007	0.013	-0.018	-0.499	0.618	
	Education level, higher education	0.002	0.034	0.002	0.070	0.945	
	Standard of living	0.061	0.025	0.083	2.486	0.013	
	Political position	0.005	0.007	0.023	0.707	0.480	
2							
	Age	0.004	0.001	0.107	2.762	0.006	
	Gender, women	0.136	0.033	0.135	4.063	0.000	
	Number of persons in the household	-0.006	0.013	-0.016	-0.436	0.663	
	Education level, higher education	0.007	0.034	0.007	0.200	0.842	
	Standard of living	0.039	0.026	0.053	1.505	0.133	
	Political position	0.006	0.007	0.027	0.819	0.413	
	Satisfaction with life	0.064	0.026	0.086	2.444	0.015	
	Self-reported health	0.016	0.025	0.025	0.644	0.520	
	Chronic conditions, yes	0.088	0.039	0.081	2.249	0.025	
	Contact with a COVID-19 patient, yes	-0.035	0.042	-0.027	-0.837	0.403	
3							
	Age	0.002	0.001	0.062	1.668	0.096	
	Gender, women	0.129	0.032	0.128	4.042	0.000	
	Number of persons in the household	-0.002	0.012	-0.005	-0.147	0.883	
	Education level, higher education	0.029	0.032	0.029	0.908	0.364	
	Standard of living	0.046	0.025	0.063	1.884	0.060	
	Political position	0.006	0.007	0.029	0.924	0.356	
	Satisfaction with life	0.067	0.025	0.090	2.709	0.007	
	Self-reported health	0.075	0.026	0.118	2.885	0.004	
	Chronic conditions, yes	0.067	0.037	0.062	1.797	0.073	
	Contact with a COVID-19 patient, yes	0.015	0.040	0.011	0.364	0.716	
	Disease threat 	0.161	0.018	0.293	8.801	0.000	
	Concern for the future 	0.015	0.021	0.022	0.697	0.486	
	Perceived infectability 	0.052	0.021	0.087	2.430	0.015	
	Return to a normal life after the pandemic	-0.030	0.015	-0.063	-2.031	0.043	
4							
	Age	0.002	0.001	0.054	1.474	0.141	
	Gender, women	0.133	0.031	0.132	4.258	0.000	
	Number of persons in the household	0.001	0.012	0.002	0.066	0.947	
	Education level, higher education	0.023	0.032	0.022	0.715	0.475	
	Standard of living	0.034	0.024	0.046	1.392	0.164	
	Political position	0.005	0.007	0.024	0.764	0.445	
	Satisfaction with life	0.046	0.025	0.062	1.866	0.062	
	Self-reported health	0.068	0.025	0.107	2.668	0.008	
	Chronic conditions, yes	0.056	0.037	0.052	1.523	0.128	
	Contact with a COVID-19 patient, yes	0.012	0.040	0.009	0.291	0.771	
	Disease threat 	0.132	0.019	0.240	7.099	0.000	
	Concern for the future 	0.018	0.021	0.027	0.865	0.387	
	Perceived infectability 	0.053	0.021	0.089	2.537	0.011	
	Return to a normal life after the pandemic	-0.036	0.015	-0.075	-2.434	0.015	
	Trust in medical and scientific institutions 	0.140	0.030	0.179	4.755	0.000	
	Trust in the Swiss government 	0.019	0.028	0.025	0.690	0.490	
	Trust in non-Swiss institutions 	0.005	0.022	0.008	0.233	0.816	


S3. Detailed multiple linear regression analysis with effectiveness of othering strategies overall (Trust, disease threat and protective measures, Switzerland, 2020).	
Model	Unstandardized Coefficients	Standardized Coefficients	t	P Value	
	B	Std. Error	Beta			
1							
	Age	0.010	0.002	0.156	4.436	0.000	
	Gender, women	0.055	0.057	0.030	0.959	0.338	
	Number of persons in the household	-0.008	0.022	-0.012	-0.361	0.718	
	Education level, higher education	-0.204	0.058	-0.111	-3.541	0.000	
	Standard of living	-0.164	0.042	-0.122	-3.898	0.000	
	Political position	0.111	0.012	0.284	9.271	0.000	
2							
	Age	0.009	0.002	0.146	4.012	0.000	
	Gender, women	0.057	0.057	0.031	1.001	0.317	
	Number of persons in the household	-0.004	0.022	-0.005	-0.156	0.876	
	Education level, higher education	-0.205	0.058	-0.112	-3.559	0.000	
	Standard of living	-0.136	0.045	-0.101	-3.046	0.002	
	Political position	0.112	0.012	0.286	9.347	0.000	
	Satisfaction with life	-0.070	0.045	-0.051	-1.560	0.119	
	Self-reported health	-0.018	0.043	-0.016	-0.424	0.672	
	Chronic conditions, yes	0.111	0.067	0.056	1.657	0.098	
	Contact with a COVID-19 patient, yes	-0.119	0.072	-0.050	-1.652	0.099	
3							
	Age	0.007	0.002	0.111	3.123	0.002	
	Gender, women	0.047	0.056	0.025	0.839	0.402	
	Number of persons in the household	0.002	0.022	0.003	0.087	0.930	
	Education level, higher education	-0.173	0.056	-0.094	-3.100	0.002	
	Standard of living	-0.125	0.043	-0.093	-2.893	0.004	
	Political position	0.113	0.012	0.288	9.606	0.000	
	Satisfaction with life	-0.065	0.043	-0.048	-1.508	0.132	
	Self-reported health	0.074	0.045	0.064	1.632	0.103	
	Chronic conditions, yes	0.078	0.065	0.039	1.193	0.233	
	Contact with a COVID-19 patient, yes	-0.046	0.070	-0.019	-0.648	0.517	
	Disease threat 	0.230	0.032	0.229	7.220	0.000	
	Concern for the future 	0.020	0.037	0.017	0.550	0.583	
	Perceived infectability 	0.090	0.037	0.082	2.419	0.016	
	Return to a normal life after the pandemic	-0.048	0.026	-0.055	-1.838	0.066	
4							
	Age	0.007	0.002	0.115	3.205	0.001	
	Gender, women	0.047	0.056	0.026	0.849	0.396	
	Number of persons in the household	0.002	0.022	0.003	0.081	0.935	
	Education level, higher education	-0.176	0.056	-0.096	-3.136	0.002	
	Standard of living	-0.121	0.043	-0.090	-2.792	0.005	
	Political position	0.111	0.012	0.283	9.310	0.000	
	Satisfaction with life	-0.057	0.044	-0.042	-1.291	0.197	
	Self-reported health	0.080	0.045	0.069	1.762	0.078	
	Chronic conditions, yes	0.072	0.066	0.036	1.095	0.274	
	Contact with a COVID-19 patient, yes	-0.041	0.071	-0.017	-0.575	0.566	
	Disease threat 	0.245	0.033	0.243	7.403	0.000	
	Concern for the future 	0.018	0.037	0.015	0.492	0.623	
	Perceived infectability 	0.091	0.037	0.083	2.445	0.015	
	Return to a normal life after the pandemic	-0.046	0.026	-0.053	-1.770	0.077	
	Trust in medical and scientific institutions 	-0.001	0.052	-0.001	-0.026	0.979	
	Trust in the Swiss government 	-0.050	0.049	-0.037	-1.023	0.307	
	Trust in non-Swiss institutions 	-0.035	0.039	-0.030	-0.890	0.374	
